# Safe Skin Management during Open Hepatectomy in a Patient with Recessive Dystrophic Congenital Epidermolysis Bullosa

**DOI:** 10.1155/2018/1786786

**Published:** 2018-06-28

**Authors:** Ikuo Watanobe, Hiroko Kida, Yuuki Sekine, Masaya Kawai, Shozo Miyano, Michio Machida, Toshiaki Kitabatake, Hiroyuki Sugo, Yoshifumi Lee, Kuniaki Kojima

**Affiliations:** ^1^Department of General Surgery, Juntendo University Nerima Hospital, 3-1-10 Takanodai, Nerima-ku, Tokyo 177-8521, Japan; ^2^Department of Nursing, Juntendo University Nerima Hospital, 3-1-10 Takanodai, Nerima-ku, Tokyo 177-8521, Japan

## Abstract

Congenital epidermolysis bullosa is a rare, genetic condition in which even slight stimulation can cause blistering of the skin or mucosa. While previous reports of treatments requiring general anesthesia in these patients were focused on anesthesia-related procedures, such as endotracheal intubation, no report has described specific management required for these patients during surgery, such as preparation of the surgical site, fixation of infusion lines and other tubes, and adjustment of the operation table. This is probably the first report to address these issues. This report presents a case of recessive dystrophic congenital epidermolysis bullosa in which open hepatectomy was safely performed.

## 1. Introduction

Congenital epidermolysis bullosa (EB) is a rare group of diseases in which even slight mechanical stimulation can cause blistering of the skin and mucosa. Open surgery used to be rarely performed in EB patients, since endotracheal intubation was contraindicated in these patients due to the risk of blistering of the tracheal mucosa and subsequent postoperative complications [1–3]. On the other hand, as reported by Jemes and Wark [4] and Tomlinson [5], no complication has been reported in EB patients who actually underwent endotracheal intubation. We safely performed hepatectomy under general anesthesia in a patient with recessive dystrophic epidermolysis bullosa (RDEB), the severest form of EB. This is the first report to describe specific skin management procedures during open surgery in an EB patient.

## 2. Case Report

The patient was a 65-year-old man with a prior history of repeated plastic surgery for scar contracture of the hands and fingers, ablation surgery for idiopathic ventricular tachycardia, and diabetes. There was no family history of no consanguineous marriage or EB.

He had experienced recurrent blistering of the skin that was readily caused by an external force since the time shortly after birth, which had been treated symptomatically. He was diagnosed with EB during a genetic consultation that he had received before getting married at the age of 28 years. Subsequently, he was diagnosed with RDEB at the age of 38 years. Application of a strong external force to the skin results in blister formation as early as 15 min. In June 2012, he presented to a nearby hospital with epigastric pain, where he was diagnosed with cholelithiasis and cancer in the transverse colon and was referred to our hospital. In September 2012, transverse colectomy and cholecystectomy were performed via laparotomy, followed by an uneventful postoperative course. In April 2013, a liver metastasis (S2) was detected. The lesion was a solitary tumor measuring ≤2 cm and was treated by radiofrequency ablation (RFA) in June 2013, again followed by an uneventful postoperative course. In September 2015, a recurrent tumor was detected at the site of RFA, with suspected invasion into the diaphragm. He was then admitted to our hospital for curative open surgery. On admission, although no active blistering was noted, pigmentation and scars due to recurrent blistering were noted especially in the extremities and back. Most fingers in both hands were club-shaped, with a few intact fingers. Blood test showed a mild increase in glucose to 123 mg/dl and increases in tumor markers, including mean levels of CEA and CA19-9 of 25.0 (0–5) ng/ml and 62.1 (0–37) U/ml, respectively.

Abdominal CT/MRI revealed a 3.5 cm metastatic liver carcinoma with diaphragmatic invasion in the lateral segment of the liver. In January 8, 2016, open partial hepatectomy of the lateral segment with combined diaphragmatic resection was performed.

### 2.1. Surgical Management

The patient was asked to climb on the operating table on his own to minimize application of an external force to the skin. Epidural anesthesia was achieved by just one injection of 5 ml of 0.5% procaine into the epidural space. For endotracheal intubation, due to a difficulty in manually fixing a mask and lifting the lower jaw, the patient was asked to open his mouth and intubation was performed while the patient was conscious using intravenous injection of 1% propofol and intratracheal spraying of 1% xylocaine, under bronchoscopic guidance using a McGRATH™ MAC video laryngoscope (Covidien). Isodine disinfectant was used for skin disinfection of the surgical site, as the patient was tolerant of chemical stimulations. A skin incision was made sharply with a scalpel, with particular care taken to avoid contact of a steel instrument with the skin. Partial hepatectomy of the lateral segment with combined diaphragmatic resection was performed. The diaphragmatic defect was closed with a 2-0 nonabsorbable suture while the lung was compressed, without chest tube placement. A 19 Fr closed low-pressure continuous-suction drain was placed on the liver resection surface. A block catheter was also placed on the bilateral rectus sheaths in case of postoperative wound pain. The wound was closed by two-layer suturing with a 0 monofilament absorbable suture for the peritoneal muscle layer and a 4-0 monofilament absorbable suture for dermal closure. The wound was covered with a Mepilex® Border Ag dressing (Mölnlycke Health Care). The drain was fixed with a needle and a suture and then with a Mepitac® tape (Mölnlycke Health Care). The operative and anesthetic times were 346 and 457 min, respectively.

The patient was discharged from the hospital on day 9. He had an uneventful postoperative course with no abnormality of the wound in postoperative outpatient examination.

## 3. Discussion

EB was first reported by Hebra [6] in 1870 as Erblichen Pemphigus. Subsequently, Köbner defined the condition as epidermolysis bullosa in 1886 [7]. Fine et al. [8] reported the incidence of the condition to be 1 out of every 50,000 births and classified it into 4 major types according to clinical features, site of blister formation, and hereditary form, including (1) simple, (2) junctional, (3) dominant dystrophic, and (4) recessive dystrophic types. The 4 types are further classified into 16 subtypes [9]. The present case was the recessive dystrophic type, which is characterized by the most pronounced and variable clinical manifestations, including severe scar formation, tooth hypoplasia, ankylodactyly, esophageal stenosis, pyloric atresia, and other causes of gastrointestinal obstruction. Studies examining the association of EB with malignancy [10–12] have shown that 5–10% of all cases of RDEB are associated with squamous cell carcinoma, which is considered to arise from scars secondary to recurrent blistering. In particular, the extremities are the major sites of origin of basal cell carcinomas and squamous cell carcinomas [13, 14].

Several reports have described the pathophysiology of RDEB, cicatrization leading to finger/toe deformities, and general anesthesia for RDEB patients [15]. Meanwhile, no report has described the details of major open surgery involving EB patients. General anesthesia is associated with thermal burns due to inappropriate management, skin disorder and nerve injury caused by compression, and other problems, even in non-EB patients. Extra attention is needed for RDEB patients in whom application of even a slight external force can result in blister formation as features of the condition.

**Figure 1:**
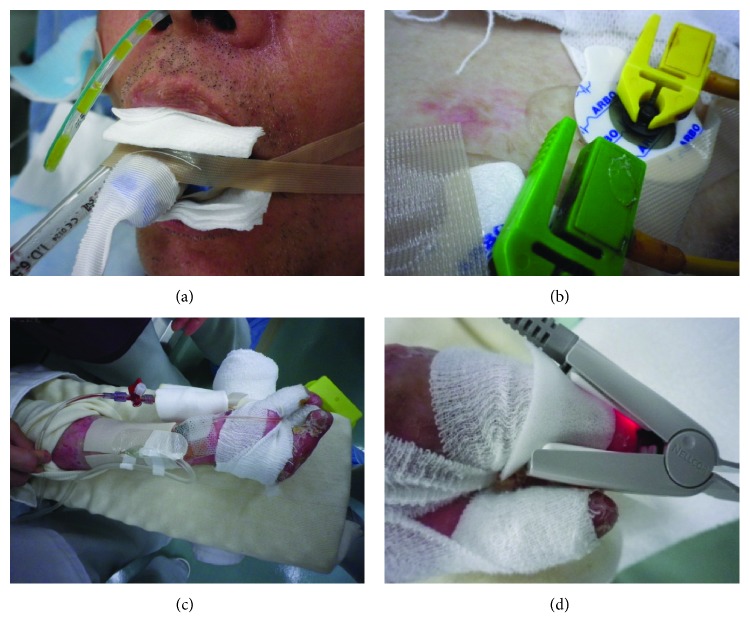
Fixation of infusion lines, tubes, and vital sign monitoring devices. (a) A Mepilex Lite dressing (Mölnlycke Health Care) and gauze were placed between the lips and a tracheal tube and fixed with a Mepitac tape (Mölnlycke Health Care). (b) An ECG electrode was placed on a conductive high-viscosity gel and fixed with a Mepitac tape. (c) An infusion tubing was fixed to the skin by suturing, with Mepilex Lite placed between the tubing and skin. (d) A clip-type pulse oximeter was used with Mepilex Lite placed between the device and skin.

**Figure 2:**
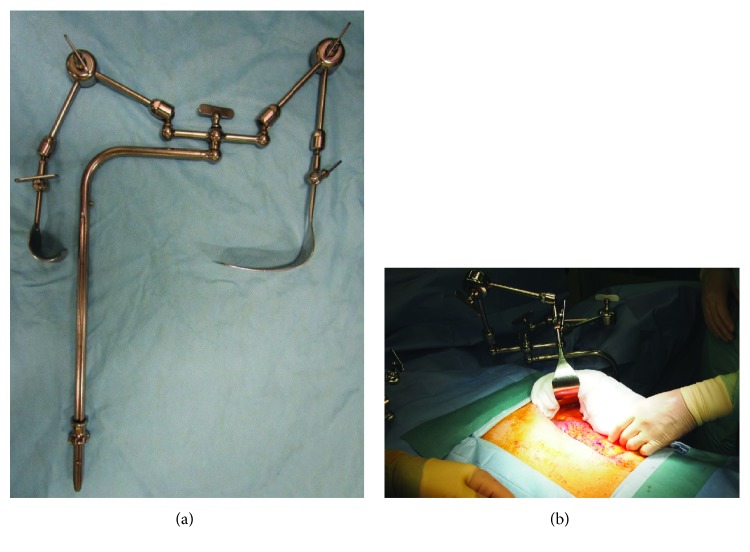
This retractor is characterized by the three-joint arm structure that allows for fixation of the device in a free position, rather than retracting in just one direction. The surgical site was prepared, avoiding stress to the skin by lifting a skin flap in the vertical direction, rather than opening the wound in the horizontal direction.

**Figure 3:**
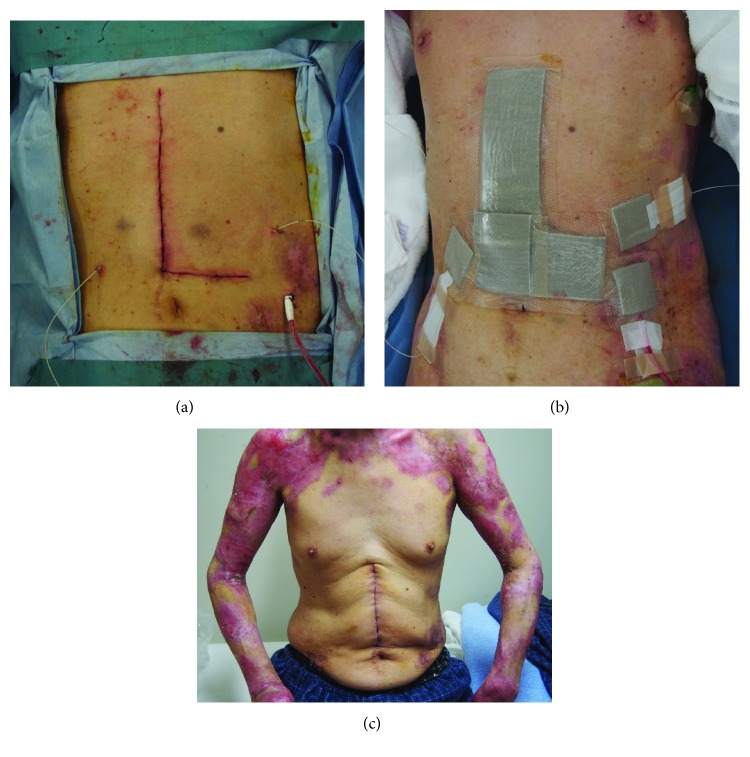
Postoperative appearance of the skin. (a) A picture taken immediately after the operation, showing no blistering around the wound, drain insertion site, or block catheter insertion sites on the bilateral rectus sheaths. (b) The wound was protected with a Mepilex Border Ag dressing (Mölnlycke Health Care), with a Mepilex Lite dressing placed between the tubes and skin to fix the tubes. (c) A picture taken after discharge (2 weeks later). The patient had an uneventful postoperative course with no blister formation.

The present patient underwent upper abdominal laparotomy followed by hepatectomy, which required a sufficient size of abdominal incision and extended surgical management. The operating table was covered by artificial fat (Action® O.R. Overlay; Action Products Inc.), a body-pressure dispersion mat (SOFT NURSE; TT SAFE Med ApS), and a waterproof sheet, which were layered in this order. Since regular adhesive tapes could not be used, venous and arterial lines were all fixed with a needle and a suture. For fixation of tubes and electrodes, Mepitel® (Mölnlycke Health Care) or Mepilex Lite (Mölnlycke Health Care) were placed between these tubes/electrodes and skin, followed by fixation with wound dressing/protection materials coated entirely with silicone, such as a Mepilex Border Ag dressing (Mölnlycke Health Care) and a Mepitac tape (Mölnlycke Health Care) (Figure 1). For the skin incision, a large, L-shape incision was made in the upper abdomen to minimize the external force caused by an abdominal retractor (OCTOPUS retractor holder® with OCTOPUS Liver Retractor®; Yufu Itonaga Co. Ltd.) (Figure 2). With these efforts, the operation was completed without new blister formation (Figure 3).

The same management was applied to the patient when he underwent open transverse colectomy plus cholecystectomy for a cancer of the transverse colon and cholelithiasis in September 2012 and laparoscopic RFA (pneumoperitoneum pressure: 10 cmH_2_O, pneumoperitoneum time: 94 min) in June 2013, allowing for the operation to be completed safely without new blister formation in any part of the body, including the surgical site.

## 4. Conclusion

We experienced a valuable case in which laparotomy was performed safely without any complication in a patient with RDBE, the severest form of EB.
